# Editorials

**Published:** 1897-08

**Authors:** 


### HOMŒOPATHIC PHYSICIAN,

A MONTHLY JOURNAL OF

HOMŒOPATHIC MATERIA MEDICA AND CLINICAL MEDICINE.

"If our school ever gives up the strict inductive method of Hahnemann, we are lost, and deserve only to be mentioned as a caricature in the history of medicine."—CONSTANTINE HERING.

Vol. XVII. AUGUST, 1897. No. 8.

### EDITORIALS.

CALCAREA-CARBONICA.—One of the indications for Calcarea is the open condition of the fontanelles. This makes us think, too, of Calc-phos., Silicea, Sulphur.

Sensation on the right side of the head as if a piece of ice were lying there. This is a great characteristic of Calcarea. Cold spots here and there on the body is always an indication for Calcarea. Coldness of the vertex is an indication for Veratrum. Calcarea has itching and stinging in the corners of the eyes. Painful sensitiveness of eyes, as if from a foreign body in the eyes. There is burning and cutting in eyes and eyelids.

Calcarea has agglutination of eyelids at night with redness and swelling. Aconite and Lycopodium have agglutination of the eyes at night, and watering during the day. Pulsatilla has agglutination of eyes at night, preventing the patient opening them on waking in the morning until they are washed.

Calcarea has oozing of blood from the eye and Belladonna from the ear.

Calcarea has twitching of upper or of lower eyelid. Calcarea patients are far-sighted.

Calcarea, like Belladonna, is indicated in inflammation of ears.

Calcarea has cracking in ears when eating. It is a great remedy for polypus in the ear and in the nose. Like Belladonna, Calcarea is a great remedy for inflammation of the parotid gland.

Calcarea has inflamed, swollen, red nose, and Belladonna has erysipelas beginning upon the nose. The nose of Calcarea is dry at night and watery during the day. It is sometimes stuffed with yellow fetid pus.

Foul odor from the nose is a characteristic of Calcarea. There is entire loss of smell and taste under this remedy. Diminished sense of smell is an indication for Calcarea and for Natrum-muriaticum as well. Calcarea has pale, bloated face with blue rings around eyes. Calcarea has emaciation of the face and Natrum-muriaticum emaciation of the neck. Calcarea has eruptions on lower lip and Lycopodium on the upper lip. Calcarea has pain in face followed by swelling of the cheek. There may be swelling of the whole face.

An indication for Calcarea is swelling of upper lip, though Apis, Belladonna, Bovista, and Staphysagria have it.

Calcarea is useful in difficult dentition of children, and so is Silicea. Calcarea is indicated in fistulæ of the gums leading to the roots of carious teeth. This is similar to Fluoric-acid and Silicea. Calcarea has sour taste in the mouth with white coated tongue. Food tastes as if not salted enough. Puls., food tastes too salty.

Spasmodic contractions of œsophagus indicate both Belladonna and Calcarea; inflammatory swelling of palate, with blisters upon it indicates both Calcarea and Kali-bichrom.

Calcarea has thirst with loss of appetite. This is like Sulphur. Calcarea has hunger soon after eating. Calcarea has great desire for eggs; this is especially true in scarlet fever. The Calcarea patient is much troubled with water brash. The patient has aversion to coffee. Calcarea has nausea from drinking cold water. It also has bitter eructations. Calcarea has pressure and cramp pain in stomach, with vomiting after dinner. This pressure is worse from the clothing bound around the waist. Nitric-acid has amelioration from tight clothing. Calcarea has enlargement of abdomen from swelling of the mesenteric glands. This is similar to Silicea and sugar. Calcarea has aggravation from drinking water of all affections of the stomach. Calcarea has white stools. Guernsey's key-note is "clay-colored stools." It is one of the most reliable indications in the materia medica. The diarrhœa of Calcarea is generally painless. Calcarea has protrusion of hemorrhoids during stool, with burning pain. Calcarea is an excellent remedy in prolapsus ani. It is indicated for burning hemorrhoids, and differs from Apis, which has burning, *stinging*, hemorrhoids. Calcarea has incontinence of urine. Calcarea is useful in polypus of bladder.

---

Hering on Typhoid Fever.—In the June number the editor expressed the hope that Dr. Charles B. Gilbert would yet be able to finish his intended appendix to Hering's book.

Dr. Gilbert desires the editor to request of the members of the profession to send contributions of their own clinical experience in the strictly homœopathic treatment of typhoid fever, so that he may be enabled to make the repertory as full as possible. "If we can once get the united experience of the whole profession we can have a work that will last a long time."

As Dr. Bell says in his preface to the fourth edition of his famous monograph on diarrhœa, "Homœopathy is not making that kind of 'progress' that renders a whole medical library obsolete every ten years." Hence Dr. Gilbert's desire to incorporate the "united experience of the whole profession" will have the effect of making a work of incalculable value.

